# Quantitative Evaluation of the Compatibility Effects of Huangqin Decoction on the Treatment of Irinotecan-Induced Gastrointestinal Toxicity Using Untargeted Metabolomics

**DOI:** 10.3389/fphar.2017.00211

**Published:** 2017-04-21

**Authors:** Dong-Ni Cui, Xu Wang, Jia-Qing Chen, Bo Lv, Pei Zhang, Wei Zhang, Zun-Jian Zhang, Feng-Guo Xu

**Affiliations:** ^1^MOE Key Laboratory of Drug Quality Control and Pharmacovigilance, China Pharmaceutical UniversityNanjing, China; ^2^State Key Laboratory of Natural Medicine, China Pharmaceutical UniversityNanjing, China; ^3^State Key Laboratory for Quality Research in Chinese Medicines, Macau University of Science and TechnologyMacau, China

**Keywords:** Huangqin decoction, delayed diarrhea, metabolomics, metabolites deregulation score, relative area under the curve

## Abstract

Huangqin decoction (HQD), a traditional Chinese medicine (TCM), has been widely used to treat gastrointestinal syndrome in China for thousands of years. Chemotherapy drug irinotecan (CPT-11) is used clinically to treat various kinds of cancers but limited by its side effects, especially delayed diarrhea. Nowadays, HQD has been proved to be effective in attenuating the intestinal toxicity induced by CPT-11. HQD consists of four medicinal herbs including *Scutellaria baicalensis* Georgi, *Glycyrrhiza uralensis* Fisch, *Paeonia lactiflora* Pall, and *Ziziphus jujuba* Mill. Due to its complexity, the role of each herb and the multi-herb synergistic effects of the formula are poorly understood. In order to quantitatively assess the compatibility effects of HQD, mass spectrometry-based untargeted metabolomics studies were performed. The serum metabolic profiles of rats administered with HQD, single *S. baicalensis* decoction, *S. baicalensis*-free decoction and baicalin/baicalein combination were compared. A time-dependent trajectory upon principal component analysis was firstly used to visualize the overall differences. Then metabolites deregulation score and relative area under the curve were calculated and used as parameters to quantitatively evaluate the compatibility effects of HQD from the aspect of global metabolic profile and the specifically altered metabolites, respectively. The collective results indicated that *S. baicalensis* played a crucial role in the therapeutic effect of HQD on irinotecan-induced diarrhea. Both HQD and SS decoction regulated glycine, serine and threonine pathway. This study demonstrated that metabolomics was a promising tool to elucidate the compatibility effects of TCM or combinatorial drugs.

## Introduction

Huangqin decoction (HQD) has been widely used to treat gastrointestinal syndrome in China for thousands of years. Irinotecan (CPT-11) is used clinically to treat various kinds of cancers (Ikuno et al., 1995; Souglakos et al., 2006; Kummar et al., 2011) but limited by its side effects like diarrhea, including short duration early secretory diarrhea and life-threatening delayed diarrhea (Sparreboom et al., 1998; Santos et al., 2000; Slatter et al., 2000; Xie et al., 2002). Nowadays, HQD (Wang et al., 2015) and its modified pharmaceutical preparation PHY906 (Farrell and Kummar, 2003; Kummar et al., 2011) have been proved to be effective in attenuating the intestinal toxicity induced by CPT-11 in preclinical and clinical trials.

Huangqin decoction consists of four medicinal herbs including *Scutellaria baicalensis* Georgi, *Glycyrrhiza uralensis* Fisch, *Paeonia lactiflora* Pall, and *Ziziphus jujuba* Mill. It is believed that the interaction between herbs could lead to synergistic effects (Cheng, 2015). For HQD, researches have revealed that *Angelica dahurica* extracts had synergistic action on *S. baicalensis* by enhancing the intestinal absorption of its major bioactive component baicalin *in vivo* (Liang et al., 2012). This herbal pair also showed synergistic effect on each other in relieving oxidative stress (Yang et al., 2011). Furthermore, *S. baicalensis* could work synergistically with *Coptis chinensis* or *Pinellia ternata* to get more rapid dissolution of the effective ingredients (Takasuna et al., 1995; Feng et al., 2008).

Although it has been confirmed that multi-components would interacted with multi-targets and finally led to synergistic effects in most formulas, the detailed potential mechanisms are still ambiguous. Due to the complexity of chemical composition, only using a single index or a simple superposition method could not be feasible to unveil the multi-herb synergistic characteristics of TCM (Jin et al., 2016). Thus, recently, researchers tried to use knockout/knockin chromatography, bioactive equivalence oriented feedback screening method (Song et al., 2016) and system pharmacology strategy (Huang et al., 2014; Zhang W.J. et al., 2016) to recognize the bioactive equivalent combinatorial components (BECCs) or the most effective chemicals in one formula. As a novel holistic approach to study metabolic alternations, metabolomics was also introduced to study the complex herbal prescriptions (Pelkonen et al., 2012). However, how to quantitatively assess the efficacy of herbal drugs based on metabolomics is still challenging. Most metabolomics studies only compared the separation among control, disease and therapy groups based on PCA score plots visually, and without quantitative basis (Zhang et al., 2010; Li et al., 2016). In order to translate the direct phenotypic graph to quantifiable data, relative mean distance values (RDV) of different groups in PLS-DA score plots were calculated and used to quantitatively assess drug toxicity (Aa et al., 2010). However, the RDV are largely dependent on the number of principal components. A recently proposed method, *Pathifier* (Drier et al., 2013), which has been successfully used in genomics to quantify the pathway deregulation level in a sample for cancer early diagnosis and stratify (Drier et al., 2013; Livshits et al., 2015; Huang et al., 2016) showed its practicability.

In this study, in order to assess the compatibility effects of HQD on the treatment of irinotecan-induced diarrhea quantitatively, liquid chromatograph-mass spectrometer and gas chromatograph-mass spectrometer based untargeted metabolomics studies were performed. The efficacy effects of HQD, single *S. baicalensis* decoction, *S. baicalensis*-free decoction or baicalin/baicalein combination on the treatment of irinotecan-induced diarrhea were evaluated. The overall perturbations observed in serum metabolic profiles were compared. Two parameters, MDS and RAUC, were used to quantitatively evaluate the compatibility effects of HQD from the aspect of global metabolic profile and the specifically altered metabolites, respectively.

## Materials and Methods

### Chemicals and Reagents

Irinotecan hydrochloride (CPT-11) was obtained from Hengrui (Jiangsu, China). Four component herbs of HQD, *Scutellaria baicalensis* Georgi (Hebei province), *G. uralensis* Fisch (Inner Mongolia of China), *P. lactiflora* Pall (Anhui province), and *Z. jujuba* Mill (Henan province) were purchased from Tongrentang drugstore (Nanjing, China) and authenticated by Dr. Wei Zhang (State Key Laboratory for Quality Research in Chinese Medicines, Macau University of Science and Technology, China). Baicalin, baicalein, methoxyamine hydrochloride, *N*-methyl-*N*-trifluoroacetamide (MSTFA) and pyridine were purchased from Sigma–Aldrich (St. Louis, MO, USA). Methanol, acetonitrile and ethyl acetate for high-performance LC-grade were obtained from Honeywell (Burdick and Jackson, NJ, USA). Deionized water was purified by a Milli-Q system (Millipore, Bedford, MA, USA).

### Decoction Preparation

The dry herb pieces of *S. baicalensis* Georgi (dried roots) (9 g), *P. lactiflora* Pall (dried roots) (6 g), *G. uralensis* Fisch (dried and honey-fried roots and rhizomes) (6 g), and *Z. jujuba* Mill (dried fruits) (6 g) were extracted twice with boiling water (1:15 and then 1:10) for 1.5 and 1 h and filtered through gauze. Then the merged mixtures were concentrated to dryness by a 2.5 L freeze dry system (Labconco, USA). Finally, suspension was prepared by adding 27 ml water (1 g/mL) for intragastric administration (i.g.). Single *S. baicalensis* and *S. baicalensis*-free decoctions were prepared through the same procedure with the equal weights of medicine. Besides, according to the ratio in HQD, BB were mixed (13.6:2.5) mg/mL and suspended in water to prepare for the bioactive constituents.

### Animal Study and Sampling

Sixty-three male Sprague-Dawley rats (weighing 200 ± 20g) were purchased from the Sino-British SIPPR/BK Lab Animal Ltd. (Shanghai, China). All animals were kept in a standard animal laboratory with regulating temperature and humidity (20–22°C, 45 ± 5%), and 12/12 h light-dark cycle, with free access to chow and water. All experiments were authorized by the Animal Ethics Committee of China Pharmaceutical University and carried out under the Guidelines for Care and Use of Laboratory Animals.

After 1-week accommodation, the rats were randomly divided into six groups: CPT-11 alone (T), HQD treatment (T/HQD), Single *S. baicalensis* decoction treatment (T/SS), BB combination treatment (T/BB), *S. baicalensis*-free decoction treatment (T/SF) and control (C). Normal saline or CPT-11 diluted with it was administered intravenously (i.v.) to the animals *via* the tail vein with 150 mg/kg/day of body weight from days 1 to 2. Treatment groups were intragastrically administrated with HQD, SS decoction, BB combination, or SF decoction from day 0 (about 0.5 h before administration of CPT-11), twice each day for continuous 7 days. Oral and intravenous administration schedules and dosages were shown in **Table 1**.

**Table 1:** Oral and intravenous administration schedule and dosage of each group.

Group	T/HQD	T/SS	T/BB	T/SF	T	Control
Rats number	12	10	10	10	11	10
i.v. Schedule	CPT-11	CPT-11	CPT-11	CPT-11	CPT-11	Normal saline
i.g. Schedule	HQD^a^	SS decoction	BB combination^b^	SF decoction	Water	Water
Lavage dosage	10 g/kg	3.3 g/kg	B: 136 mg/kg BG: 25 mg/kg	3.3 g/kg	Equivalent water	Equivalent water

*^a^10 g/kg: 10 g crude herbs per 1 kg rat body weight. ^b^Dosage of baicalin and baicalein corresponding to content of them in HQD, in the form of co-administrated mixture. HQD, Huangqin decoction; SS, single *S. baicalensis*; BB, baicalin and baicalein; SF, *S. baicalensis* free; T, CPT-11; i.g., intragastric; i.v., intravenous.*

Body weight and diarrhea score (DS) were monitored twice per day (before oral gavage) from days -1 to 13. Rats feces were scored using the following scale: 0, normal (normal stool or absent); 1, slight diarrhea (wet and soft stool); 2, moderate diarrhea (wet and unformed stool with moderate perianal staining of the coat); 3, severe diarrhea (watery stool with severe perianal staining of the coat) (Kurita et al., 2000). As shown in Supplementary Figure S1, blood samples from retinal venous plexus were collected at days -1, 1, 4, 7, and 10, then clotted, centrifuged at 8000 rpm for 10 min, and stored at -80°C until GC/MS and LC/MS analysis. Two rats from each group were euthanized by day 4. The jejunum, ileum, cecum and colon were collected to prepare paraffin section and perform hematoxylin-eosin staining for histological examination.

### Sample Preparation and Metabolomic Analysis

Sample preparation and analytical method of instruments were based on our previous studies (as shown in Supplementary Information) (Wang et al., 2015; Dai et al., 2016). All tested samples were analyzed randomly. LC/MS analysis was performed on a Shimadzu Prominence series ultrafast liquid chromatography (UFLC) system coupled with ion trap/time-of-flight hybrid mass spectrometry (IT-TOF/MS) (Shimadzu Inc., Kyoto, Japan). The column for separation was Phenomenex Kinetex C18 (100 mm × 2.1 mm, 2.6 μm) (Phenomenex, Torrance, CA, USA). GC/MS analysis was performed on Shimadzu GCMS-QP2010 Ultra (Ultra GC-Q/MS; Shimadzu Inc., Kyoto, Japan) equipped with an Rtx-5MS capillary column (30.0 m × 0.25 mm ID, 0.25 μm).

### Data Processing and Statistical Analysis

Peak detection and alignment were performed by profiling solution version 1.1 (Shimadzu, Kyoto, Japan) to get the matrix of peak intensities from the original LC-MS and GC-MS chromatographs, respectively. Following background-peak-filtering, 80 rule, limitation of QCs, normalization by the total ion intensity, ion fusion, and Pareto scaling (Dai et al., 2016; Zhang P. et al., 2016), PCA and OPLS-DA (orthogonal projection to latent structures-discriminant analysis) were conducted by SIMCA-P (version 13.0, Umetrics, Sweden). Parameters of PCA (R^2^X and *Q*^2^) and OPLS-DA (R^2^X, R^2^Y and *Q*^2^) were used to evaluate the models. Besides, variable importance in the projection (VIP) values in the OPLS-DA model were used to select potential biomarkers. Additionally, Mann–Whitney *U* test was performed in MeV (version 4.6.1^1^) and *p*-values were adjusted by false discovery rate correction based on the Benjamini-Hochberg method (Broadhurst and Kell, 2006).

### Biomarkers Screening and Identification

For each time point, OPLS-DA models were constructed between group T and other groups, respectively. Features with VIP > 1.0 and *p* < 0.05 between the two groups were regarded as potential biomarkers and selected for identification.

For LC/MS datasets, peaks were preliminarily identified by referring to online databases (HMDB, METLIN, and Lipid MAPS) or related literatures (ppm < 30). Furthermore, the preliminarily identified metabolites were determined by comparing with the reference standards available in our lab or the fragment models in databases. For GC/MS datasets, metabolites were located in the original chromatographs and preliminarily identified by NIST 11 (National Institute of Standards and Technology). Some important biomarkers were further confirmed by reference standards available in our lab. The potential metabolites were connected by MetaboAnalyst for pathway analysis^2^.

### Data Quality Evaluation in Metabolomics Analysis

For validating the stability of the analysis process, quality control samples (QCs) were first prepared by mixing serum from each test sample (10 μL aliquot). QCs were injected ten times before the batch process and injected one time every eight samples during the analysis process, to monitor the stability of sample preparation and instrument.

The procedure of removing substances originating from the decoctions is as follows. First, combining groups C and T into one group, called CT group. The OPLS-DA model between T/HQD and CT was constructed. Then, trend plots of features in S-plot were shown, features absent in CT were the components from decoction that need to be removed (Liu and Yan, 2013).

### Compatibility Effects Evaluation Methods

**FIGURE 1:**
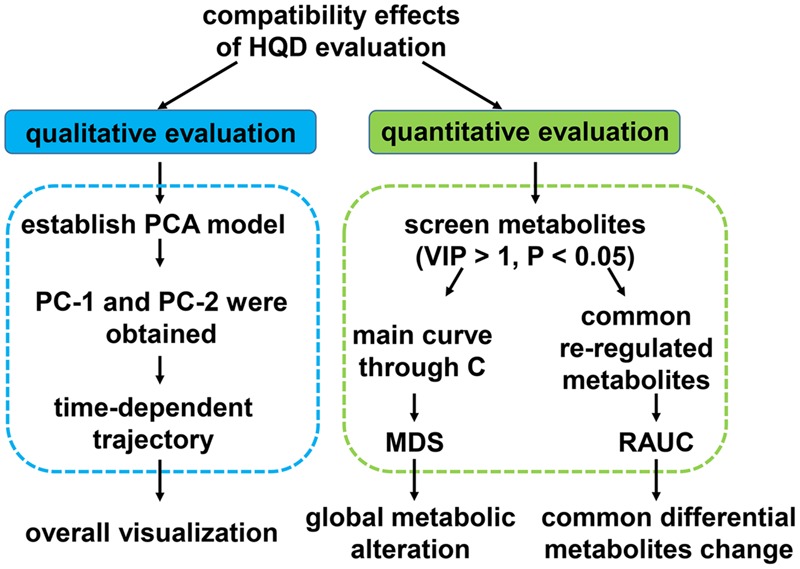
**Scheme of compatibility effects evaluation**.

After serum metabolomics analysis, GC/MS data, LC/MS (+) and LC/MS (-) data were integrated as the whole dataset, all the efficacy evaluations were based on whole dataset. Scheme of compatibility effects evaluation was showed in **Figure 1**.

First, PCA model based on whole dataset was established. Average value of the first or the second principle components (PC1 and PC2) of every group at each time point was calculated. Each dot represents the average metabolic status at a certain time. They were plotted on one graph with PC1 as *X*-axis, PC2 as *Y*-axis and then different group was curved on separate graph so that the results could be displayed more clearly.

Second, R package *pathifier* was applied to perform metabolites set analysis (Drier et al., 2013). All of the samples dataset were looked as a cloud. We built up the “principal curve” through healthy samples in the cloud. The “principal curve” was firstly put forward as a non-parametric non-linear extension of the linear PCA. It passed through the middle of the data to provide a non-linear summary of the data (Hastie and Stuetzle, 1989). Due to the limited metabolic pathway coverage of untargeted metabolomics approach, we chose to calculated the overall MDS instead of pathway deregulation score (PDS) using the R package *pathifier* (Huang et al., 2016). Every sample was projected onto the principle curve and it’s MDS was the projection distance value of each sample. The level of sample metabolites deregulation was quantified by MDS. Area under the MDS curves were also calculated.

Third, common re-regulation metabolites in T/HQD, T/SS, and T/BB three groups, T/HQD and T/SF two groups were under investigation. Fold change values relative to group control at each time point compose the relative curves. RAUC from days 4 to 10 was calculated. The lower the RAUC level, the sample is more similar to control.

## Results

### Body Weight and Histopathology Examination

**FIGURE 2:**
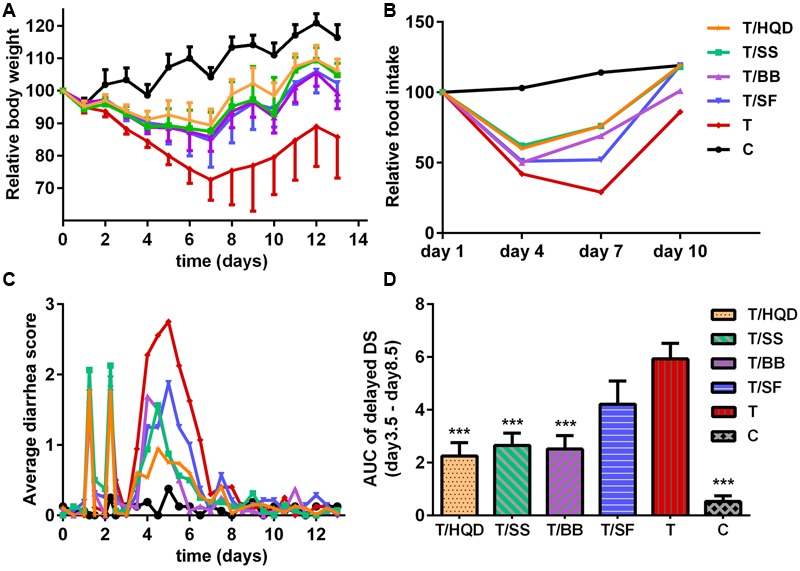
**Effect of TCM (HQD, SS, BB, or SF decoctions) on CPT-11-induced toxicity in rats. (A)** Effects of TCM in protecting against weight loss induced by CPT-11. The change in body weight was calculated on the basis of that on day –1. Each point represents the mean of 8–10 animals. **(B)** Effects of TCM in protecting against food-intake reduce induced by CPT-11. **(C)** Effects of TCM in protecting against diarrhea induced by CPT-11. **(D)** Area under the curve (AUC) of delayed diarrhea score (DS) (days 3.5–8.5). ^∗∗∗^*p* < 0.001 vs. T group. Details of experimental procedures are given in section “Materials and Methods.” SS, single *S. baicalensis*; BB, baicalin and baicalein; SF, *S. baicalensis* free; T, CPT-11.

**FIGURE 3:**
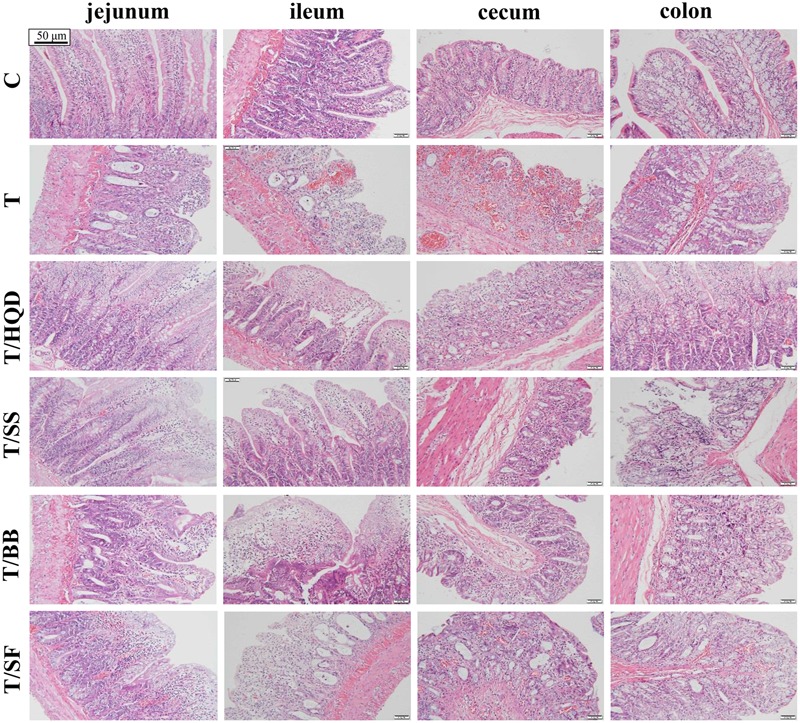
**Effect of TCM (HQD, SS, BB, or SF decoctions) on CPT-11 induced intestinal damage in rats.** Hematoxylin and eosin (H&E) staining was used to visualize the formalin-fixed sections of jejunum, ileum, cecum, and colon 3 days after treatment with CPT-11. SS, single *S. baicalensis*; BB, baicalin and baicalein; SF, *S. baicalensis* free; T, CPT-11.

Comparing to the group C, the relative body weight (RBW), DS and its AUC, reduction of food intake and histopathology examination of group T indicated that CPT-11 induced serious delayed diarrhea which mainly lasted from days 4 to 5. The results were consistent with our previous research (Wang et al., 2015) (**Figures 2A–D, 3**).

Meanwhile, the serious delayed diarrhea was partly alleviated in decoction treated groups (T/HQD, T/SS, T/BB, and T/SF) with different degrees. Taken the DS, RBW, incidence of severe diarrhea and histological examination in day 4 and/or 5 into account, the treatment effects of these groups were sorted as HQD > SS decoction > BB combination > SF decoction (**Table 2**). These results were indicative, but not conclusive since the difference of treatment effect among these decoctions were not significant. Additionally, DS and histological examination was not so objective and RBW was not so closely related with serious delayed diarrhea.

**Table 2:** Effect of TCM treatment on the CPT-11-induced delayed diarrhea.

Group	Average diarrhea score (D4-5) ±*SE*^a^	Relative body weight (D5/D0%) ±*SE*^b^	Incidence of delayed diarrhea (D4-5) (%)^c^	
			Score 2 and 3	Score 3
Control	0.17 ± 0.08	107.12 ± 1.37	3.13	0
T/HQD	0.74 ± 0.19***	92.71 ± 1.35***	10.00	0
T/SS	0.98 ± 0.18**	89.32 ± 1.77**	18.75	0
T/BB	1.14 ± 0.21**	88.69 ± 1.46**	34.38	6.25
T/SF	1.41 ± 0.34*	89.14 ± 1.94**	53.13	6.25
T	2.43 ± 0.12###	72.17 ± 8.06###	77.50	42.50

*^a^Average score of delayed diarrhea was calculated for each animal by counting observations with scores from days 4 to 5. ^b^Relative body weight at day 5 was calculated for each animal relative to animal’s weight at the beginning of the study (day -1). ^c^Incidence of DSs 2 and 3 and incidence of DS 3 were calculated for each animal by counting observations with that score two consecutive days (4 and 5). ^∗^*p* < 0.05, ^∗∗^*p* < 0.01, ^∗∗∗^*p* < 0.001 vs. T group; ^###^*p* < 0.001 vs. control group. HQD, Huangqin decoction; SS, single *S. baicalensis*; BB, baicalin and baicalein; SF, *S. baicalensis* free.*

### Data Quality Evaluation in Metabolomics Analysis

Aiming to detect as many endogenous metabolites as possible, full scan mode was performed in sample analysis which meant all substances in serum were detected. So, the exogenous components derived from HQD were also included in the dataset. In order to eliminate the influence of those exogenous components, chemical constituents originating from the HQD were recognized and removed first. Taken the constituents in HQD as an example, OPLS-DA was conducted between non-decoction treated groups (C and T) and HQD treated group (R^2^X = 0.767, R^2^Y = 0.971, *Q*^2^ = 0.766). S-plot and trend plot were used to screen out decoction-originated compounds. For example, peak 102 was filtered out by S-plot, and trend plot demonstrated it was originated from HQD (Supplementary Figure S2). Finally, 53 and 159 peaks from GC/MS and LC/MS were retained, respectively.

As shown in Supplementary Figure S3, QC samples were close in PCA score plots. Furthermore, the retention time shift was less than 0.1 min and the RSD values of all peaks in QCs were lower than 15%, which demonstrated the whole analytical process was stable and reliable (Naz et al., 2014).

### Time-dependent Trajectory Based Qualitative Evaluation

**FIGURE 4:**
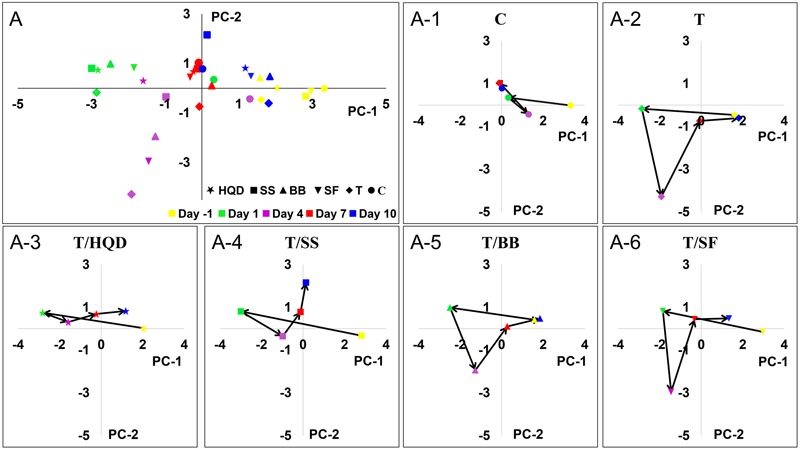
**The time-dependent trajectory of the metabolic profiles of control, T and TCM (HQD, SS, BB, or SF decoctions) treatment groups.** Each dot represents the average metabolic status at a certain time. **(A)** Summary graph. **(A-1)** to **(A-6)** The trajectory change for each group. SS, single *S. baicalensis*; BB, baicalin and baicalein; SF, *S. baicalensis* free; T, CPT-11.

To construct a model containing overall metabolites information, the datasets from LC/MS (ESI+), LC/MS (ESI-) and GC/MS were combined and analyzed by a PCA model. In the PCA model of all test samples (R^2^X = 0.919, *Q*^2^ = 0.591), the trajectory change of group C in different days was relatively small, while the deviation in group T was obvious. Comparing with day -1 in group T, the distances of other days were correlated to DS and RBW: the distance of day 4 (i.e., the day of delayed diarrhea) was the longest, then it was greatly reduced at day 7 and finally it was close to zero at day 10 (**Figure 4**). Also, the results of PCA indicated the differences between group C and group T at days 4, 7, and 10 were related to the delayed diarrhea (Supplementary Figure S3). Meanwhile, the trajectory analysis indicated the differences between group C and decoction-treated groups during delayed diarrhea were less obvious than that between group C and group T.

Particularly at day 4, the deviations of group T/HQD and T/SS were much less than that in group T. OPLS-DA score plots of day 4 showed the metabolic profiles of group T/HQD, T/SS and T/BB were separated from group C and group T, while group T/SF were mixed with group T (Supplementary Figure S4). These results visualized the time-dependent trajectory of CPT-11 exposure and decoction treatment effect from the aspect of global metabolic alternation.

### Quantitative Evaluation of the Compatibility Effects

Using the biomarkers selection method described above, totally 47 discriminating metabolites (VIP > 1 and *p* < 0.05) were screened out and identified. Based on Spearman correlation analysis between altered metabolites and DS/RBW, four metabolites were removed because of the weak correlation (-0.5 < Spearman correlation coefficients < 0.5) (Supplementary Figure S5). The remaining 43 metabolites were used to calculate MDS.

**FIGURE 5:**
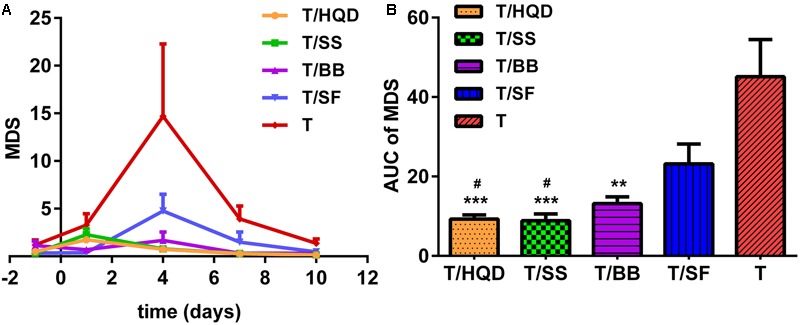
**Relative quantitation of TCM efficacy based on MDS. (A)** Time dependent MDS curves of CPT-11 and TCM (HQD, SS, BB, or SF decoctions) treatment groups. **(B)** Area under the curve (AUC) of MDS curves. ^∗∗^*p* < 0.01, ^∗∗∗^*p* < 0.001 vs. T group; ^#^*p* < 0.05 vs. SF group. SS, single *S. baicalensis*; BB, baicalin and baicalein; SF, *S. baicalensis* free; T, CPT-11.

Principal curve and data-cloud of samples were shown in Supplementary Figure S6. In general, the MDS demonstrated that the CPT-11 induced metabolites deregulation on day 1, further (the most) on day 4, and then the abnormalities were almost recovered on days 7 and 10. While decoction-treated groups (T/HQD, T/SS, T/BB, and T/SF) were closer to control. Furthermore, MDS curves and the areas under the curves (**Figures 5A,B**) evaluated the treatment effects of decoctions quantitatively. At day 4, group T, T/SF, or T/BB were significantly different (*p* < 0.05) from group T/HQD and T/SS. However, groups T/HQD and T/SS had no significant difference between each other (Supplementary Figure S7).

On the overall MDS basis, we further compared difference between decoction treatment groups and group T based on re-regulation metabolites. The relative concentration of creatine, creatinine, glycine, LysoPE (20:4), proline, valine, nonanedioic acid, acetylcarnitine, citric acid and methionine were commonly re-regulated in T/HQD, T/SS, and T/BB treatment groups. Creatine, creatinine, glycine, LysoPE (20:4), proline, valine, nonanedioic acid and alanine were re-regulated in both T/HQD and T/SF treatment groups. Creatine, creatinine, glycine, LysoPE (20:4), proline and valine are the common re-regulation metabolites.

**FIGURE 6:**
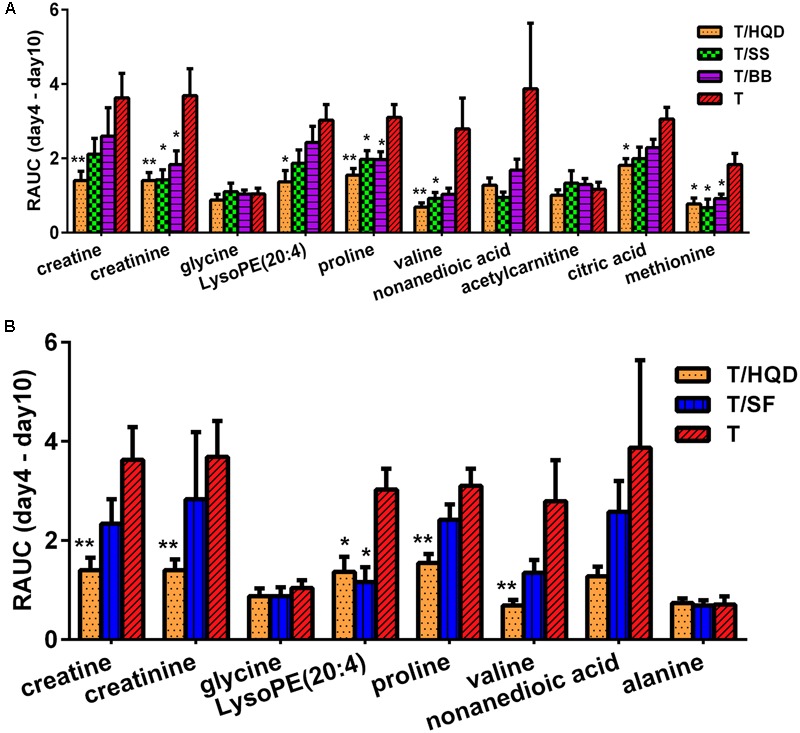
**Re-regulated metabolites’ RAUC from days 4 to 10. (A)** T/HQD, T/SS, T/BB vs. T. **(B)** T/HQD, T/SF vs. T. (Control is the zero level of *Y*-axis). ^∗^*p* < 0.05, ^∗∗^*p* < 0.01 vs. T group [not false discovery rate (FDR)-adjusted *p*-values]. SS, single *S. baicalensis*; BB, baicalin and baicalein; SF, *S. baicalensis* free; T, CPT-11.

Relative area under the curve results demonstrated that groups T/HQD, T/SS and T/BB had obvious variance. For group T/HQD, RAUC of creatine, creatinine, LysoPE (20:4), proline, valine, citric acid and methionine were significantly lower than that of group T. As to group T/SS, RAUC of creatinine, proline, valine, and methionine were significantly lower than group T. Except for valine, other re-regulated metabolites in the T/BB group were the same with T/SS group (**Figure 6A**). In addition, five common re-regulated metabolites were significantly restored in group T/HQD (**Figure 6B**). However, only LysoPE (20:4) was significantly restored in group T/SF.

All above results indicated that CPT-11 induced distinct change of metabolites in days 4–10, and groups T/HQD, T/SS, and T/BB showed retracement trend. Moreover, group T/HQD showed the most obvious trend of retracement in common metabolites. Using RAUC as an indicative parameter, the difference in treatment effect of each TCM on CPT-11 induced intestinal toxicity could be quantified from the aspect of the specific change of individual differential metabolite.

### Pathway Analysis

After biomarkers identification and spearman correlation analysis, 43 metabolites were reserved. All of the reserved 43 metabolites were significantly perturbed at least in 1 day during the process of CPT-11 exposure and listed in Supplementary Tables S1, S2. Heat-maps showed the time-dependent fluctuations (red = increased concentration, green = decreased concentration) of metabolites as a result of CPT-11 perturbation and decoction deregulation (Supplementary Figure S8). Except for leucine, lysine, threonine, tryptophan, glutamic acid, uric acid, glycocholic acid, linoleic acid, LysoPC (18:0), LysoPC (20:3) and LysoPE (16:0), other metabolites were re-regulated at least 1 day in decoction treatment groups (T/HQD, T/SS, T/BB, or T/SF).

**FIGURE 7:**
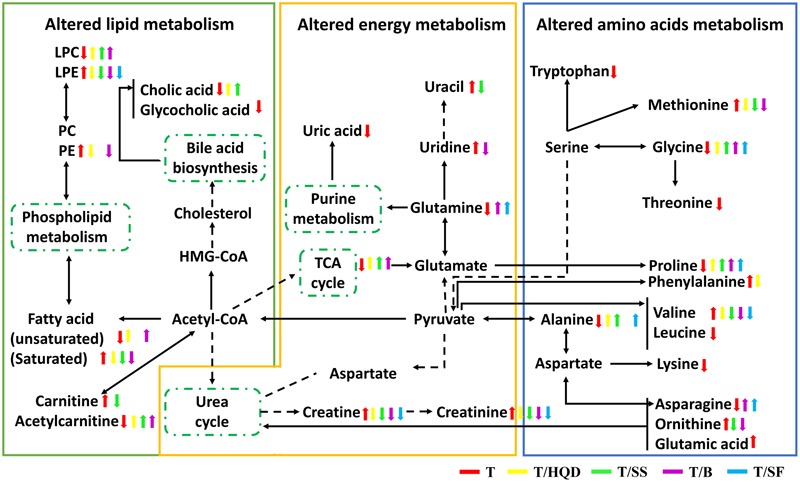
**The perturbed metabolites and metabolic pathways related to CPT-11 treatment as well as TCM (HQD, SS, BB, or SF decoctions) modification.** Elevation (up arrows) and reduction (down arrows) of the levels of metabolites observed in rats are indicated. SS, single *S. baicalensis*; BB, baicalin and baicalein; SF, *S. baicalensis* free; T, CPT-11.

It has been reported that five metabolic pathways in serum were identified associated with CPT-11 exposure (phenylalanine, tyrosine and tryptophan biosynthesis; phenylalanine metabolism; glycine, serine and threonine metabolism; alanine, aspartate and glutamate metabolism; and primary bile acid biosynthesis) and HQD attenuated the side-effects with reversed glutamine, tryptophan metabolism, and lipid metabolism (Wang et al., 2015). In the present study, D-glutamine and D-glutamate metabolism, arginine and proline metabolism, and valine, leucine and isoleucine biosynthesis pathways are also considered to be relevant pathways influenced by CPT-11. These metabolism changes are related to energy, amino acid and lipids metabolism. Furthermore, we also found that decoction treatment groups achieved a comprehensive efficacy via multiple pathways and targets (**Figure 7**). The number and extend of re-regulated metabolites in groups T/SS, T/BB, and T/SF are all less than those in group T/HQD.

It is noted that HQD alleviated the delayed diarrhea induced by CPT-11 mainly *via* modulating glycine, serine and threonine metabolism, and phenylalanine, tyrosine and tryptophan biosynthesis pathways. Besides, glycine, serine and threonine metabolism, and arginine and proline metabolism pathways play an important role in SS decoction mitigates the delayed diarrhea.

## Discussion

### Qualitative Analysis of TCM Efficacy

Traditional Chinese medicine formulas are an important source for the development of new drugs to prevent and treat gastrointestinal toxicity induced by chemotherapeutics (Liu and Cheng, 2012). In studies of HQD, its component *S. baicalensis* was described in *Bencao Gangmu* (*Compendium of Materia Medica*) about 420 years ago for the treatment of diarrhea, dysentery, and inflammation (Li, 1593/2012). Moreover, as the major bioactive constituents in *S. baicalensis*, baicalin and its aglycones baicalein can protect injured intestinal epithelial cells (Yao et al., 2016) and active autophagy in cancer cells (Li-Weber, 2009). The mechanism research further demonstrated that baicalin might be able to promote the polarization of macrophages to an M2 phenotype to relieve ulcerative colonic inflammation (Zhu et al., 2016). Afterward, other studies also shown that BB could protect intestinal epithelial cells and inhibit mTORC1 in human cancer cells (Aryal et al., 2014). And so, we think it’s reasonable to speculate that *S. baicalensis* and its active component BB (BB) have ameliorative effect on CPT-11-induced gastrointestinal toxicity. Because baicalin is absorbed as the form of baicalein and they have “enterohepatic circulation” phenomenon *in vivo* (Li et al., 2012), rats in group T/BB were administrated the mixture of BB. Mean metabolic position had been used in PCA trajectory plots to map the metabolic changes in chemicals exposure by Nicholson et al. (2002). So, we studied the serum metabolome to reveal trajectories of PCA score plot derived from C, T and TCM treatment groups.

The results showed that HQD achieved the best efficacy with alleviated intestinal tissue pathological damage, SF decoction was the lowest one, and SS decoction and BB combination were in between. The results not only supported that CPT-11 induced metabolic profiles changes in a time-dependent manner but also revealed the overall time-dependent dynamic changes of difference groups. These observations were in agreement with the theory of the compatibility of HQD.

In the development of herbal therapy, single herb formulae were gradually evolved into the multi-herb formulae. Modernization of TCM focuses on multi-components formulae (Wang et al., 2012). Consequently, the results of this study may provide some heuristic guidance for TCM formulation rationalization and optimization.

### Relatively Quantitative Analysis of TCM Efficacy Based on MDS

We developed a new method to quantitatively evaluate the efficacy of TCM referring to PDS. PDS was first proposed in genomics study, and was used to transforms gene-level information into pathway-level information for cancer stratification and diagnosis (Drier et al., 2013; Huang et al., 2016). In the light of this idea, we calculated the overall MDS instead of PDS to evaluate the level of global metabolic alteration quantitatively. Using the method presented above, we selected the best relevant features, screened significantly changed metabolites, and then calculated the overall MDS at each time points to assess the compatibility effects dynamic changes of serum profile. It is especially interesting that MDS of groups T/HQD and T/SS have no significant difference. However, the MDS of groups T/SF and T are significantly higher at day 4.

These results demonstrated that, *S. baicalensis* played a major role in anti-CPT-11 toxicity effects among the four herb constituents of HQD formula. The reason leading to this results might be that HQD’s targeted facilitated intestinal recovery function could not be reflected completely in blood (Lam et al., 2010).

As to the RAUCs of these common differential metabolites, it is especially interesting that proline, methionine and creatinine are related to T/HQD, T/SS, and T/BB three treatment groups. A recent study demonstrated that CPT-11 triggered inflammation, induced apoptosis and antioxidant, changed the profile of intestinal bacterial (Lam et al., 2010, 2014). Methionine is an endogenous antioxidant which plays an essential role in protection against oxidative damage (Luo and Levine, 2009). Other studies also revealed that methionine made a contribution to the stability of the *Escherichia coli* (Oshima and Miwa, 2016). Proline and transmembrane protein were both involved in the intestinal mucosa epithelium mucin secretion, to ensure the integrity of the mucous barrier (Wu, 1998). The gastrointestinal mucosal barrier played a crucial role in the regulation of intestinal secretion, absorption (Wu, 1998) and intestinal immunity (Wu, 2009). Serum creatinine is a traditional indicator of kidney injury (Wyss and Kaddurah-Daouk, 2000). The up-regulated of creatinine in the group T was in consistent with our previous research that CPT-11 brought out renal injury (Yao et al., 2017). Thus, we speculated that HQD, SS decoction and BB combination could contribute to maintain the stability and integrity of the intestinal tract, and mitigate the damage of tissues.

### Altered Pathways Related to HQD and SS Decoction Efficacy

Intriguingly, both HQD and SS decoction elevated the level of glycine, which belongs to glycine, serine, and threonine pathway and it is highly associated with intestinal mucosal and barrier function. It has been demonstrated that CPT-11 caused destruction of mucosal architecture, increased the number of apoptotic cell, and triggered inflammation (Lam et al., 2010).

It has been established that intestinal inflammation increased threonine utilization and caused threonine deficiency (Remond et al., 2009). Furthermore, threonine is an necessary amino acid for the mucous layer (Faure et al., 2005; Mao et al., 2011). Thus, threonine is particularly important for the maintenance of gut integrity. Glycine-serine conversion is strictly related to *N*^5^*N*^10^methylentetrahydrofolate synthesis (*N*^5^*N*^10^-methylen-FH_4_ is a coenzyme in the purine and pyrimidine synthesis process). Purine and pyrimidine are essential substances for intestinal epithelial cell proliferation and protein synthesis (Tedeschi et al., 2013; Wang et al., 2014). The content of threonine and glycine were decreased in group T and re-regulated in groups T/HQD and T/SS. These observations supported the result showed here that HQD and SS decoction alleviated the delayed diarrhea *via* modulating glycine, serine and threonine metabolism.

The previous literature has showed that both phenylalanine, tyrosine, and tryptophan biosynthesis pathway and arginine and proline metabolism pathway are related to inflammation reaction. Significant increase in serum phenylalanine and the phenylalanine-tyrosine ratio and inducible nitric oxide synthase (iNOS could catalyze the synthesis of arginine) mRNA are indicators of the inflammatory disease magnitude (Wannemacher et al., 1976; Lam et al., 2010). A significant down-regulation of phenylalanine was found only in HQD treatment group, implying the efficacy specificity of HQD probably resulted from regulated phenylalanine, tyrosine, and tryptophan biosynthesis pathway. It is worth pointing out here that, HQD also elevated content of proline although the arginine and proline metabolism pathway is one of the most important pathways regulated by SS. We speculate that SS decoction might re-regulated other metabolites in this pathway.

From the above, we could draw the conclusion that both HQD and SS decoction could protect the gastrointestinal tract by altering the glycine, serine and threonine metabolism pathway, and HQD altering the phenylalanine, tyrosine and tryptophan biosynthesis pathway specifically. Due to their multi-component and multi-targets, the potential mechanism of HQD, SS decoction in treating severe diarrhea induced by CPT-11 deserves further investigation and discussion.

## Conclusion

In this report, we proposed a method to quantitatively assess the efficacy of HQD, which would be further used to elucidate the compatibility effects of complex formulae. Time-dependent trajectory and MDS combine RAUC based quantitative evaluation all showed distinct changes in various compatibility. Collectively, these results indicated that *S. baicalensis* played a crucial role in HQD on the treatment of irinotecan-induced diarrhea and exerted nearly the same efficacy effect with HQD in the aspect of global metabolic alteration. Both HQD and SS decoction could protect the gastrointestinal tract mainly by altering the glycine, serine and threonine metabolism pathway. Our study also demonstrated that metabolomics could be served as a promising tool to elucidate the compatibility effects of TCM or combinatorial drug.

## Author Contributions

D-NC carried out most of the studies, performed statistical analysis and wrote the manuscript. XW and J-QC participated in the animal experiments. BL participated in the data processing work. WZ provided professional advices. Z-JZ and F-GX designed the study and revised the manuscript. All authors have read and approved the final version.

## Conflict of Interest Statement

The authors declare that the research was conducted in the absence of any commercial or financial relationships that could be construed as a potential conflict of interest.

## Footnotes

1 http://www.tm4.org/

2 http://www.metaboanalyst.ca/MetaboAnalyst/

## Abbreviations

AUC: area under the curve
BB: baicalin and baicalein
C: control
GC/MS: gas chromatography-mass spectrometry
HQD: Huangqin decoction
i.g.: intragastric
i.v.: intravenous
LC/MS: liquid chromatography-mass spectrometry
MDS: metabolites deregulation score
OPLS-DA: orthogonal partial least squares-discriminant analysis
PCA: principle component analysis
QC: quality control
RAUC: relative area under the curve
SF: *S. baicalensis* free
SS: single *S. baicalensis*
T: CPT-11
TCM: traditional Chinese medicine
