# Supplementary material for: Quantitative Evaluation of the Compatibility Effects of Huangqin Decoction on the Treatment of Irinotecan-Induced Gastrointestinal Toxicity Using Untargeted Metabolomics
Source: Front Pharmacol. 2017 Apr 21;8:211. doi: 10.3389/fphar.2017.00211 (PMC5399027; doi:10.3389/fphar.2017.00211)
Supplement: Supplementary file 1 [file Presentation_1.pdf]

Supplementary Information

Quantitative evaluation of the compatibility effects of Huangqin decoction on the treatment of irinotecan-induced gastrointestinal toxicity using untargeted metabolomics

Dong-ni Cui[1,2], Xu Wang[1,2], Jia-qing Chen[1,2], Bo Lv[1,2], Pei Zhang[1,2], Wei Zhang[3], Zun-jian Zhang[1,2], Feng-guo Xu[1,2*]**

[1]MOE Key Laboratory of Drug Quality Control and Pharmacovigilance, China Pharmaceutical University, Nanjing, 210009, China
[2]State Key Laboratory of Natural Medicine, China Pharmaceutical University, Nanjing, 210009, China
[3]State Key Laboratory for Quality Research in Chinese Medicines, Macau University of Science and Technology, Taipa, Macau, China

*** Correspondence**:
Zun-jian Zhang
Email: zunjianzhangcpu@hotmail.com
Feng-guo Xu
Email: fengguoxu@gmail.com

2 Materials and methods

2.3 Animal study and sampling

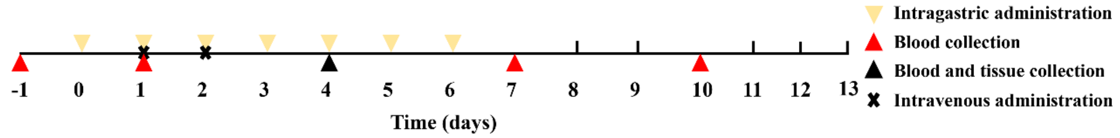

Fig. S1 Flow chart of animal experiments.

2.4 Sample preparation and metabolomic analysis

2.4.1 Sample preparation

Serum samples were thawed at room temperature. 100 µL acetonitrile and 40 µL glibenclamide (internal standard (IS), 5 µg/mL) were added to 20 µL serum and the mixture was vortexed for 5 min to extract metabolites. After centrifuged twice at 16 000 rpm (4 °C) for 10 min, the supernatant was transferred and analyzed by LC/MS.

100 mL of cold methanol (containing 5 µg/mL heptadecanoic acid, working as IS) was added to 10 µL of thawed serum and vortex-mixed for 15 min to extract metabolites. After a second centrifugation (16 000 rpm, 10 min, 4 °C), an 80 µL supernatant was obtained and transferred to a screw vial (1 mL) followed by the addition of methoxamine hydrochloride (25 µL, 10 mg/mL in dry pyridine) and incubation at 37 °C for 90 min. The mixture was evaporated to dryness and then silylated with 120 µL MSTFA/ethyl acetate (v/v, 1/1). After incubation for 2 hours at a temperature of 37 °C, the mixture was prepared for GC/MS analysis.

2.4.2 LC/MS analysis

LC/MS analysis was performed on Shimadzu ultrafast LC-ion trap time-of flight MS system equipped with an electrospray ionization (ESI) source (Shimadzu, Kyoto, Japan). Chromatographic separation was achieved on a Phenomenex Kinelex C18 column (100 × 2.1 mm, 2.6 mm, Phenomenex, Torrance, CA, USA) using a gradient elution involved 5 - 95% acetonitrile-aqueous formic acid (0.1% formic acid), 20 min; maintained with 95% acetonitrile in 3 min. The column oven was maintained at 40 °C and the flow rate of 0.4 mL/min. The ESI-MS were acquired in both positive and negative ion mode with an interface voltage of 4.5 kV and - 3.5 kV respectively. The range was scanned from 100 to 1000 m/z. The flow rate of nebulizing gas was 1.5 L/min and pressure of drying gas was 100 kPa. The temperature of heat block and curved desorption line were both 200 °C. LC/MS solution version 3.0 (Shimadzu, Kyoto, Japan) was used for mass spectra acquisition and chromatograms procession.

2.4.3 GC/MS analysis

Analysis was performed on Shimadzu GCMS-QP2010 Ultra (Shimadzu, Kyoto, Japan) equipped with a 30.0 m × 0.25 mm i.d. fused-silica capillary column with 0.25 mm Rtx-5MS stationary phase (Agilent, Shanghai, China). Helium was used as carrier gas and set at 1 mL/min. An injection volume of 1 mL was used with the split ratio of 50:1. The column temperature was initially kept at 70 °C for 3 min and then increased to 320 °C at 10 °C/min, where it was held for 2 min. The injector temperature, interface temperature and ion source temperature were set at 250 °C, 200 °C, 250 °C, respectively. Masses were acquired from m/z 45 to 600 in scan mode. The acceleration voltage was turned on after a solvent delay of 5 min. Mass spectra and chromatograms were acquired and processed with GC/MS solution version 2.7 (Shimadzu, Kyoto, Japan).

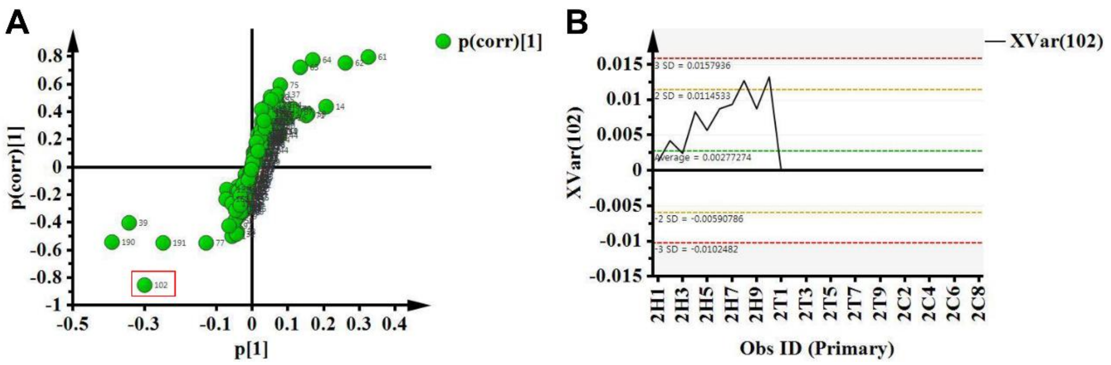

Fig. S2 Excluding chemical components come from HQD (or SS, SF decoctions). **(A)** Score plot. For example: 102 is an exogenous compound from HQD. **(B)** Trend plot of 102. SS: single *S. baicalensis*; BB: baicalin and baicalein; SF: *S. baicalensis* free; T: CPT-11.

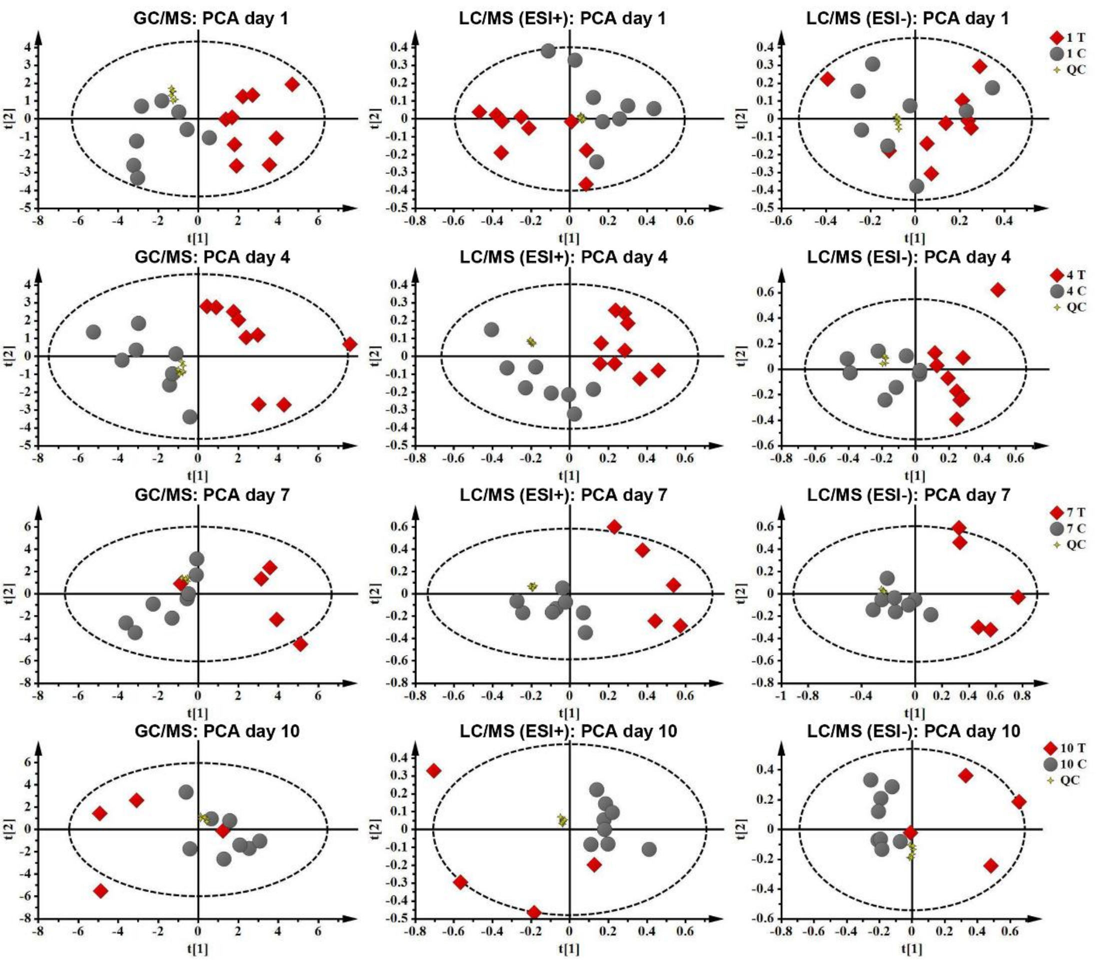

Fig. S3 PCA score plots of CPT-11 and control group at day 1, 4, 7, 10. (A)-(C): day 1; **(D)-(F):** day 4; **(G)-(I):** day 7; **(J)-(I):** day 10. (QC is quality control) Parameter of models are as follow:

model	parameter	UFLC-IT-TOF/MS (+)	UFLC-IT-TOF/MS (-)	GC/MS
Day 1	R^2X	0.787	0.812	0.753
	Q^2	0.615	0.290	0.439
Day 4	R^2X	0.772	0.678	0.763
	Q^2	0.464	0.226	0.451
Day 7	R^2X	0.779	0.771	0.694
	Q^2	0.550	0.514	0.302
Day 10	R^2X	0.877	0.736	0.726
	Q^2	0.488	0.356	0.338

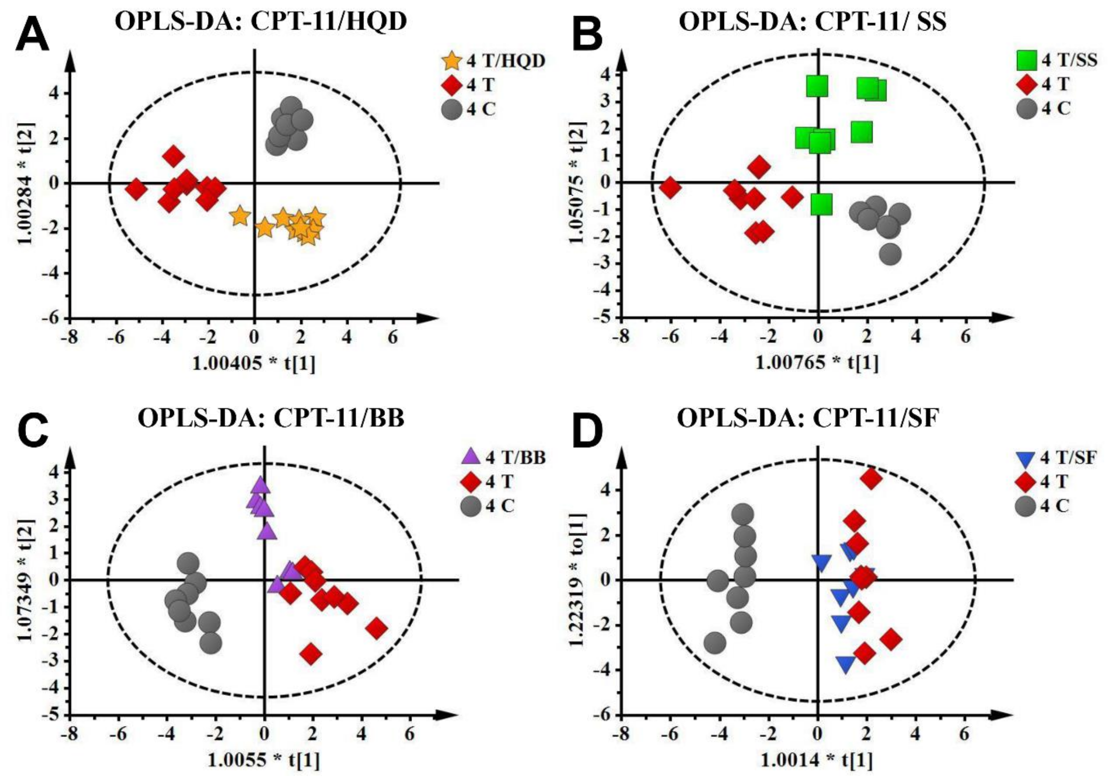

Fig. S4 OPLS-DA score plots of serum metabonomic on day 4 after CPT-11 treatment as well as TCM (HQD, SS, BB, or SF decoctions) modification. (A) HQD, $R^2X = 0.684$, $R^2Y = 0.903$, $Q^2 = 0.746$; **(B)** SS, $R^2X = 0.632$, $R^2Y = 0.765$, $Q^2 = 0.534$; **(C)** BB, $R^2X = 0.597$, $R^2Y = 0.727$, $Q^2 = 0.506$; **(D)** SF, $R^2X = 0.562$, $R^2Y = 0.502$, $Q^2 = 0.347$. SS: single *S. baicalensis*; BB: baicalin and baicalein; SF: *S. baicalensis* free; T: CPT-11.

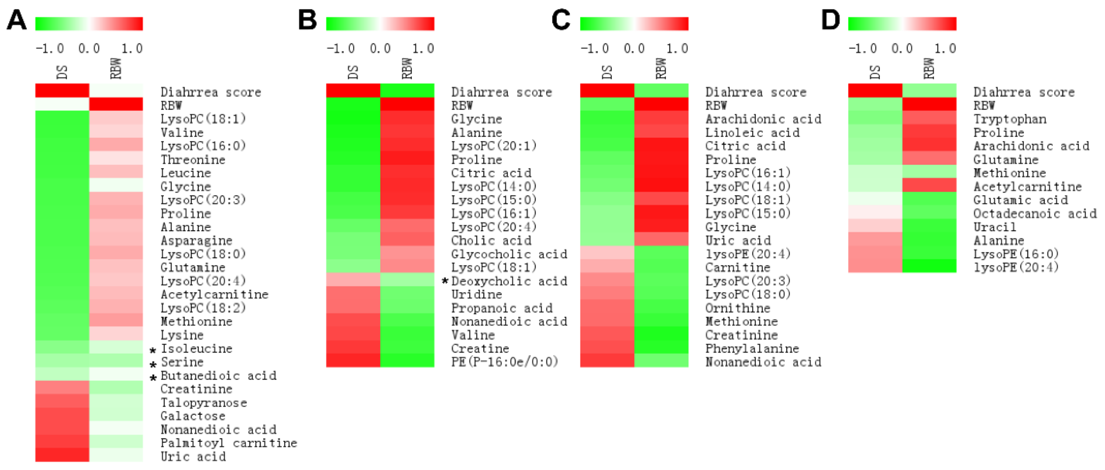

Fig. S5 Spearman correlation analysis of serum marker metabolites, diarrhea scores and body weight ratios. (A) day 1, **(B)** day 4, **(C)** day 7, **(D)** day 10. Green squares indicate significant negative correlations (- 0.5 to - 1, p < 0.05), white squares indicate nonapplicable correlations, and red squares indicate significant positive correlations (0.5 to 1, p < 0.05). (*) nonapplicable correlations metabolites.

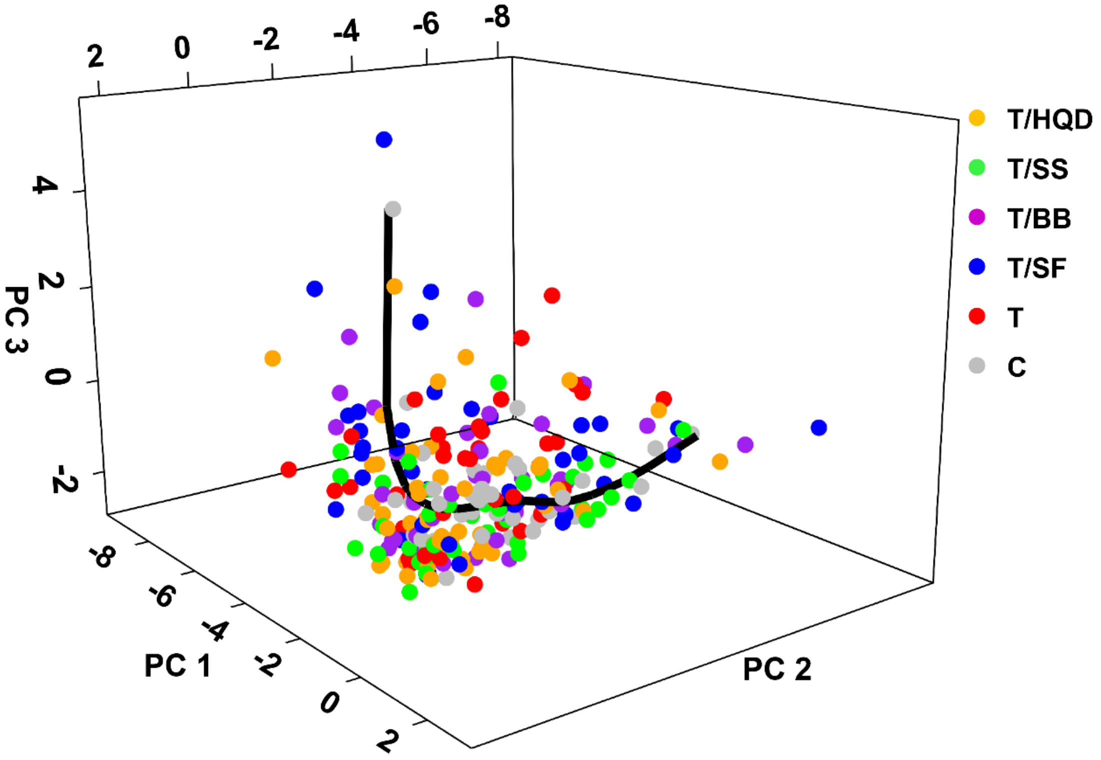

Fig. S6 The principal curve learned for the TCM (HQD, SS, BB, SF decoctions) efficacy. The principal curve (in black) going through the cloud of control samples. Other samples (from groups T/HQD, T/SS, T/BB, T/SF, C and T) projected onto the curve. SS: single *S. baicalensis*; BB: baicalin and baicalein; SF: *S. baicalensis* free; T: CPT-11.

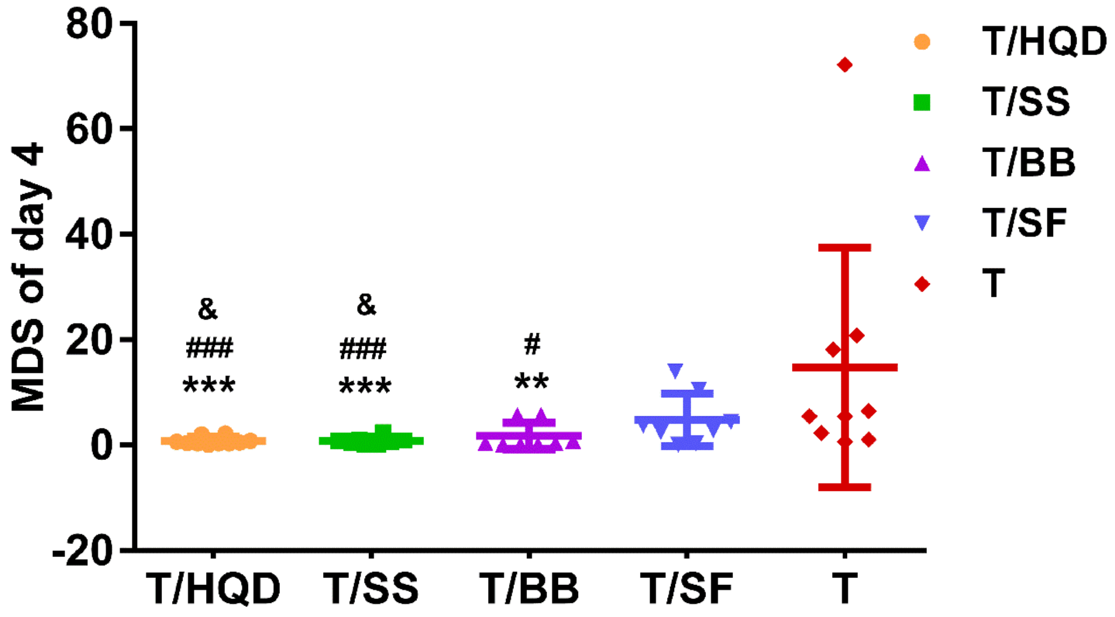

Fig. S7 MDS of groups T/HQD, T/SS, T/BB, T/SF and T at day 4. *$p < 0.05$, **$p < 0.01$, *** $p < 0.001$ vs. T group; $^{\#}p < 0.05$, $^{\#\#}p < 0.01$, $^{\#\#\#}$ $p < 0.001$ vs. SF group; $^{\&}$ $p < 0.05$, $^{\&\&}$ $p < 0.01$, $^{\&\&\&}$ $p < 0.001$ vs. BB group. SS: single *S. baicalensis*; BB: baicalin and baicalein; SF: *S. baicalensis* free; T: CPT-11.

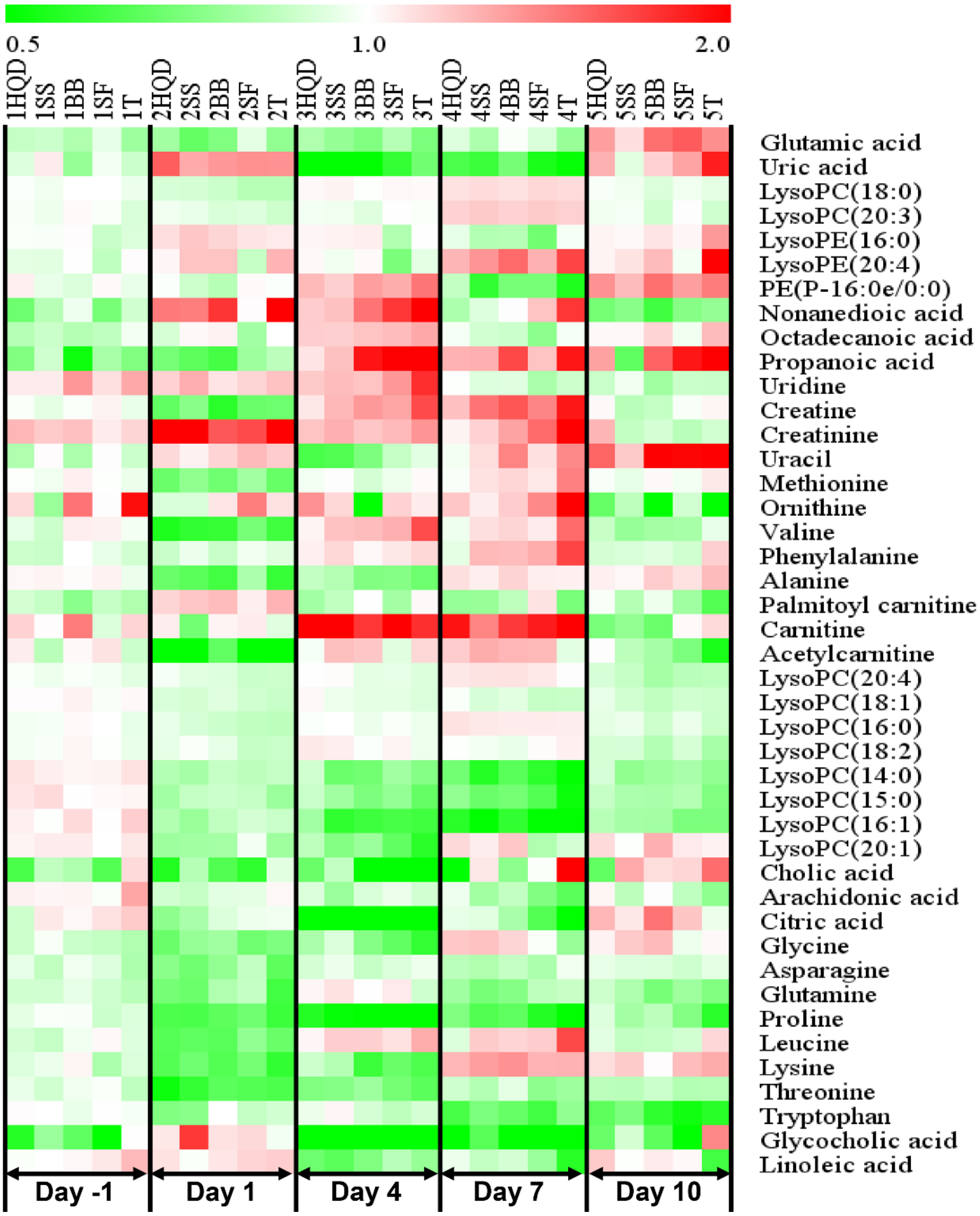

Fig. S8 Heat map of fold-changes of differential metabolites induced by CPT-11 (T group vs. Control group, p < 0.05). Each *raw* represents a metabolite feature and each *column* represents a group in one time point. The row Z-score or scaled expression value of each feature is plotted in red–green color scale (red = increased concentration, green = decreased concentration). SS: single *S. baicalensis*; BB: baicalin and baicalein; SF: *S. baicalensis* free; T: CPT-11.

Table S1 List of differential metabolites detected by GC-MS

No.	biomarker	m/z	RT(min)	Similarity [a]	VIP	p-value	r value (DS) [b]	r value (RBW) [c]
1	Alanine *	110.88	7.137	/	1.08	0.000	-0.89	0.79
2	Arachidonic acid	85.11	22.329	83	1.29	0.030	-0.77	0.77
3	Asparagine	97.41	15.325	90	1.21	0.000	-0.7	0.27
4	Citric acid	227.48	17.077	90	1.86	0.002	-0.72	0.92
5	Creatinine *	171.91	13.953	/	1.97	0.002	0.66	-0.88
6	Glutamic acid *	125.94	14.662	/	1.34	0.016	-0.07	-0.68
7	Glutamine	155.35	16.503	84	1.17	0.008	-0.35	0.57
8	Glycine	107.46	5.794	86	1.15	0.000	-0.92	0.83
9	Leucine	127.56	9.907	95	1.34	0.000	-0.74	0.26
10	Linoleic acid *	111.07	20.886	/	1.44	0.011	-0.75	0.72
11	Lysine *	174.60	15.706	/	1.26	0.015	-0.61	0.17
12	Methionine	201.12	13.425	83	2.21	0.004	0.59	-0.78
13	Nonanedioic acid	359.19	19.699	80	1.72	0.000	0.7	-0.7
14	Octadecanoic acid	245.74	21.144	92	1.05	0.016	0.06	-0.63
15	Ornithine *	70.13	14.600	/	1.84	0.019	0.58	-0.72
16	Phenylalanine	117.61	14.816	95	1.35	0.030	0.69	-0.85
17	Proline *	163.66	13.516	/	2.11	0.000	-0.85	0.9
18	Propanoic acid	110.48	6.398	96	1.83	0.006	0.57	-0.58
19	Threonine *	137.30	11.636	/	1.38	0.001	-0.74	0.12
20	Tryptophan *	238.34	21.182	/	1.61	0.008	-0.5	0.64
21	Uracil	162.45	10.924	83	2.34	0.008	0.19	-0.77
22	Uric acid *	304.67	20.045	/	1.53	0.045	-0.41	0.6
23	Uridine	131.13	18.434	80	1.24	0.002	0.57	-0.56
24	Valine *	151.35	9.035	/	1.39	0.002	0.72	-0.78

Note: * Metabolites identified by reference standards;

[a] Metabolite identified based on NIST 11, and peaks with similarity more than 80 % were assigned for compound names;

[b] Correlation coefficients of Pearson's correlation analysis between differential metabolites and DS;

[c] Correlation coefficients of Pearson's correlation analysis between differential metabolites and RBW.

Table S2 List of differential metabolites detected by LC-MS

No.	biomarker	M/Z	TR(min)	HMDB	Adduct ions	MS/MS fragment	VIP	p-value	r value (DS) [a]	r value (RBW) [b]
1	Acetylcarnitine	204.1223	0.617	HMDB00201	$[M+H]^+$	203.0515, 145.0481	2.99	0.000	-0.67	0.27
2	Carnitine	162.1123	0.616	HMDB00062	$[M+H]^+$	/	1.11	0.002	0.32	-0.67
3	Creatine	131.0694	0.653	HMDB00064	$[M+H]^+$	/	1.07	0.000	0.78	-0.76
4	Cholic acid	407.2579	9.932	HMDB00619	$[M-H]^-$	408.264, 407.2631	2.12	0.008	-0.53	0.62
5	Glycocholic acid	464.2773	8.797	HMDB00138	$[M-H]^-$	465.3042, 402.2985	1.21	0.000	-0.52	0.43
6	LysoPC (14:0)	468.3058	11.748	HMDB10379	$[M+H]^+$	450.2949, 184.0748	1.37	0.002	-0.55	0.94
7	LysoPC (15:0)	482.3211	12.517	HMDB10381	$[M+H]^+$	464.3118, 405.2478, 184.0715	1.17	0.000	-0.73	0.83
8	LysoPC (16:0)	496.3366	13.304	HMDB10382	$[M+H]^+$	478.3288, 184.0742	2.73	0.002	-0.75	0.34
9	LysoPC (16:1)	494.3208	12.207	HMDB10383	$[M+H]^+$	476.3127, 417.2412, 184.0732	2.40	0.002	-0.58	0.92
10	LysoPC (18:0)	524.3679	14.962	HMDB10384	$[M+H]^+$	506.3404, 311.2891, 184.0739	2.58	0.001	-0.69	0.33
11	LysoPC (18:1)	522.3523	13.725	HMDB02815	$[M+H]^+$	504.3431, 445.2648, 380.8514, 184.0727	1.39	0.030	-0.47	0.72
12	LysoPC (18:2)	520.3375	12.740	HMDB10386	$[M+H]^+$	502.3286, 184.0742	1.70	0.006	-0.64	0.31
13	LysoPC (20:1)	550.3830	15.303	HMDB10391	$[M+H]^+$	532.3762, 184.0742	1.22	0.000	-0.87	0.83
14	LysoPC (20:3)	546.3469	14.977	HMDB10394	$[M+H]^+$	528.3249, 184.0644	1.20	0.003	0.46	-0.64
15	LysoPC (20:4)	544.3364	12.786	HMDB10396	$[M+H]^+$	184.0723	1.11	0.046	-0.60	0.58
16	LysoPE (16:0)	454.2906	13.075	HMDB11503	$[M+H]^+$	436.2805,313.2697	1.21	0.004	0.43	-0.77
17	lysoPE (20:4)	502.2896	12.610	HMDB11517	$[M-H]^+$	303.2275, 259.2384, 205.1936	1.48	0.004	0.45	-0.94
18	Palmitoyl carnitine	400.3429	13.845	HMDB00222	$[M+H]^+$	338.3367, 239.2344, 341.2645	1.06	0.000	0.76	-0.19
19	PE (P-16:0e/0:0)	438.2968	13.530	HMDB11152	$[M+H]^+$	420.2847, 284.2948, 266.2715	1.24	0.000	0.86	-0.84

Note: [a] Correlation coefficients of Pearson's correlation analysis between differential metabolites and DS; [b] Correlation coefficients of Pearson's correlation analysis between differential metabolites and RBW.
